# A fully electronic microfabricated gas chromatograph with complementary capacitive detectors for indoor pollutants

**DOI:** 10.1038/micronano.2015.49

**Published:** 2016-02-29

**Authors:** Yutao Qin, Yogesh B Gianchandani

**Affiliations:** 1 Center for Wireless Integrated MicroSensing and Systems (WIMS ^2 ^ ), University of Michigan , Ann Arbor , MI 48109 , USA

**Keywords:** Knudsen pump, preconcentrator, separation column, gas sensor, chemical sensing, air monitoring, BTX

## Abstract

This paper reports a complete micro gas chromatography (μGC) system in which all the components are lithographically microfabricated and electronically interfaced. The components include a bi-directional Knudsen pump, a preconcentrator, separation columns and a pair of capacitive gas detectors; together, these form the *iGC3.c2* system. All the fluidic components of the system are fabricated by a common three-mask lithographic process. The Knudsen pump is a thermomolecular pump that provides air flow to the μGC without any moving parts. The film heaters embedded in the separation columns permit temperature programming. The capacitive detectors provide complementary response patterns, enhancing vapor recognition and resolving co-eluting peaks. With the components assembled on printed circuit boards, the system has a footprint of 8×10 cm^2^ . Using room air as the carrier gas, the system is used to experimentally demonstrate the analysis of 19 chemicals with concentration levels on the order of parts per million (p.p.m.) and parts per billion (p.p.b.). The tested chemicals include alkanes, aromatic hydrocarbons, aldehydes, halogenated hydrocarbons and terpenes. This set of chemicals represents a variety of common indoor air pollutants, among which benzene, toluene and xylenes (BTX) are of particular interest.

## Introduction

Volatile organic compounds (VOCs) are common contributors to indoor air pollution. The species of interest include aromatic hydrocarbons, such as benzene, toluene and xylenes (BTX), as well as alkanes, halogenated compounds, alcohols, aldehydes, ketones and terpenes. These VOCs are typically found in higher concentrations in indoor environments than outdoors ^1,2^. The exposure limits of these VOCs are typically in the range of 1-100ppm^3^. Certain species constitute severe health hazards—for example, benzene is a known carcinogen^4^.

Although VOCs can be collectively detected by a variety of standalone gas detectors, these devices typically lack the selectivity to discriminate between constituents of complex mixtures^5^. Simple mixtures can be analyzed by mass spectrometry and infrared spectroscopy systems ^6,7^, whereas complex mixtures typically require the use of gas chromatography systems ^8^. For pervasive deployment in households, offices and public facilities, microfabrication offers a pathway to reducing system cost. A system deployed for long-term indoor VOC monitoring should ideally be capable of performing automated sampling and analyses without requiring consumables or frequent replacement of components. Power consumption is not a critical concern because power sockets are widely available in indoor environments.

Since the late 1970s, a variety of micro gas chromatography (μGC) systems have been reported, in which a subset of the necessary components have been microfabricated lithographically ^9^. Most commonly, the separation column has been micromachined. Micromachined preconcentrators have been reported for sampling and injection of low-concentration VOCs into the columns^10^. However, almost all μGCs reported to date have used conventional gas valves and pumps^11-14^. Partial systems have been demonstrated using microfabricated gas pumps and conventional vapor injectors ^15^.

In addition, each of the microscale components used in these μGCs has been fabricated by separate processes. The cost and complexity of pursuing multiple microfabrication processes has constrained the implementation of fully microfabricated μGC systems. Our group has previously demonstrated an approach for fabricating all the fluidic components of a μGC using the same three-mask microfabrication process^16,17^. This approach included the integration of bi-directional gas pumps that allowed quantitative analysis without the use of the valves that are typically necessary to reverse the flow direction between sampling and separation intervals. However, our prior work used gas detectors based on microdischarge emission spectroscopy. The use of spectroscopy favors the use of a spectrometer and the use of an inert carrier gas instead of room air. A detector with direct electrical readout that can be fabricated by the fabrication process common to all other components could substantially reduce the cost and complexity of the overall system.

A number of detectors have been reported for use with μGC systems. Some have used an external optical interface^18-21^. Others that rely on an electronic detection mechanism have used a thermal conductivity detector^22^, a discharge photoionization detector^23^, a heterodyne graphene detector^24^, a surface acoustic wave (SAW) detector^11^, a metal oxide semiconductor (MOX) detector^12^, a chemiresistor^13^ or capacitive detectors^25-27^. Whereas many detectors are compatible with the use of air as carrier gas, the first three in the list of electronic detectors rely on a supply of helium as either a carrier gas or auxiliary gas. Within the context of the stated goals of micro gas chromatograph systems, capacitive detectors benefit from a commercially available, low-cost chip-scale electronic interface and compatibility with a variety of simple fabrication processes.

Of the capacitive detectors that have been reported in the past, one approach has involved the use of a parallel plate structure. One implementation^25,26^ features a polymer film sandwiched between a flat bottom electrode plate and a perforated upper electrode plate that allows vapor to reach the polymer. A more common approach has been to use interdigitated electrodes on a substrate covered by a polymer film ^27-30^. The exposed polymer undergoes both swelling and changes in electrical permittivity. Both responses affect the capacitance. However, as elucidated in this work, these responses can be utilized in ways that are complementary and consequently can be used to separate certain co-eluting species. In contrast to parallel plate electrodes, interdigitated planar electrodes provide a greater surface area over which the sampled vapor can interact with the polymer, which generally leads to a faster response. In addition, the thickness of the polymer can be varied in the final step, after lithographic fabrication is completed.

This paper reports a full μGC system, the *iGC3*.*c2* system, that integrates a microfabricated pump, a preconcentrator, separation columns and a pair of capacitive detectors that provide distinguishable responses to permittivity changes and swelling. The integrated pump is a Knudsen pump that operates by thermal transpiration^31^; it requires no moving parts, thereby providing high reliability. The preconcentrator uses a cantilever structure that alleviates thermal stress. A pair of separation columns use a non-polar stationary phase to separate BTX and other VOCs. All the components are fabricated using a common three-mask process that is a simplified version of a process described in our prior work^16^. The resulting system is a complete, easily manufacturable μGC that does not rely on external actuators, optical components, or carrier gas supply—the type of system well suited for long-term, continuous, automated monitoring of indoor pollutants. Preliminary results have been previously reported in the form of a conference abstract ^32^.

The design of the system and various components is described in Introduction, the fabrication is described in Materials and Methods, the experimental results are presented in Results, and a discussion and conclusions are presented in Discussion.

## Materials and methods

### Design

**Figure 1:**
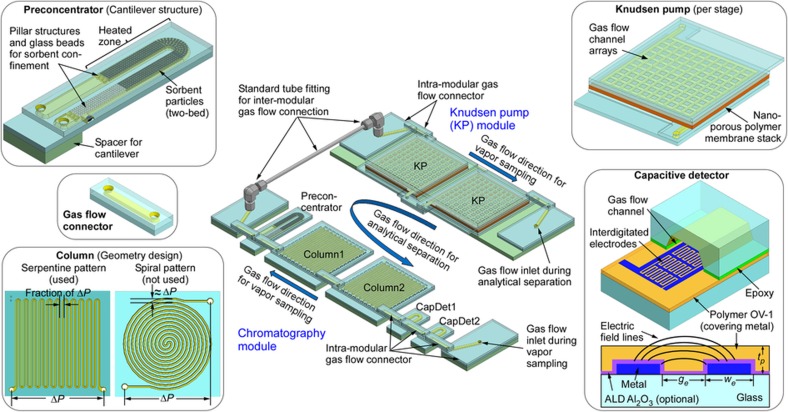
*iGC3.c2* system architecture and component design.

The *iGC3*.*c2* system is composed of a chromatography module and a pump module (Figure 1). The chromatography module features microfabricated chips that include a two-stage preconcentrator, two separation columns and two complementary capacitive detectors. The pump module includes only a two-stage, bi-directional Knudsen pump. Gas is pumped against a temperature gradient established across a mesoporous polymer material within which transport is limited to free-molecular or transitional flow regimes by the small pores^31,33^. The modules can be readily reconfigured with more separation columns and more Knudsen pump stages.

During vapor sampling, the Knudsen pump provides negative pressure to the chromatography module, drawing the vapor analytes into the preconcentrator through the capacitive detectors and separation columns. During analytical separation, the Knudsen pump provides positive pressure, reversing the flow through the chromatography module. The preconcentrator is then used to thermally inject the analytes, which are separated by the columns and detected by the capacitive detectors. The columns can be selectively heated to accelerate the separation. Embedded thin-film metal heaters are used to heat the Knudsen pump, preconcentrator and separation columns, whereas embedded thin-film metal thermistors are used to monitor the temperatures of these components.

In the preconcentrator, the sorbent particles, which absorb VOCs, are held in a channel with an elongated 'U' shape, of 1350 μm width and 300 μm height (Figure 1). The sorbent particles are confined to the lower half of the 'U' shape by pillar-like structures and glass beads that are packed against the pillars. A thin-film metal heater is located in this region to provide localized heating for vapor desorption. The gas flow inlet and outlet are located at the upper end of the 'U' shape, which is cantilevered above a glass spacer, thermally isolating it from the substrate. This design relieves thermal stress and reduces heat loss. Two segments of sorbents, Carbopack-B and Carbopack-X (Sigma Aldrich, St Louis, MO, USA), are packed in series into the 'U'-shaped channel to target a wide range of vapor species. Other types of sorbents can also be used, depending on the specific applications.

The separation columns are serpentine channels with a hydraulic diameter of 230 μm and a length of 30cm per chip. The interior of these channels is coated with a 0.2-μm-thick OV-1 stationary phase (that is, 100% polydimethylsiloxane or PDMS, Ohio Valley Specialty, OH, USA). In this work, two identical column chips were used, providing a total length of 60cm. The microfabricated columns are typically implemented in one of two shapes: a serpentine pattern and a spiral pattern. Whereas it has been reported that the serpentine pattern provides slightly higher performance than the spiral pattern^34^, in this work the serpentine pattern was used mainly because it provides a more uniform pressure distribution and a better immunity to leakage than does the spiral pattern (Figure 1). With a given pressure (Δ*P*) applied between the two ends of a serpentine column, the pressure is evenly distributed from one end of the chip to the other end. Therefore, the maximum pressure between any two adjacent channels is only a fraction of Δ*P*. In the spiral column, however, the pressure between two adjacent channels varies from zero in the chip center to Δ*P* near the chip perimeter. Therefore, any potential leak site near the chip perimeter could directly shunt the column (that is, causes gas flow to pass through the leak site but not the rest of the column), greatly compromising the performance of the separation column.

The capacitive detectors are formed using closely spaced interdigitated electrodes (Figure 1). The electrodes are covered by a layer of OV-1, which is also used as the stationary phase in the separation columns. Upon exposure to VOCs, this polymer undergoes changes in both thickness and permittivity. In principle, detector performance can be improved by downscaling various feature dimensions. Because the detector output signal is capacitance change (Δ*C*), a smaller interelectrode gap provides a higher Δ*C*. A smaller electrode width permits a denser integration of electrodes, which also provides a higher Δ*C* per unit area. In addition, a thinner polymer layer provides a faster response because the detector response time is mainly constrained by the speed of vapor absorption and desorption in the polymer layer. The interdigitated electrodes have widths and gaps of 1 μm because of the lithographic limits of the equipment available for this project. Two detectors with different polymer thicknesses are connected in series along the fluidic path, downstream of the separation column. The first detector, CapDet1, uses an OV-1 layer with a thickness of 0.25μm. Because this dimension is substantially smaller than the interelectrode gap, the electric field lines extend beyond the thickness of the polymer. Its capacitance change, Δ*C1*, is always positive, dominated by the swelling response of OV-1. The second detector, CapDet2, uses an OV-1 layer with a thickness of 1.6 μm. Its capacitance change, Δ*C2*, can be either positive or negative, depending on the dielectric constant of the vapor species relative to that of OV-1. Whereas the swelling response of the OV-1 layer does not contribute to Δ*C2*, the change in permittivity caused by the penetration of the gas molecules has a strong effect because the electric field lines are mostly contained within the polymer^30^. In each detector, the electrodes occupy a footprint of 1 mm^2^, which is enclosed in a gas flow channel with a width of 800μ m and a height of 300 μm.

The Knudsen pump module is implemented in two stages connected in series, which allows for a higher output pressure to be achieved in the gas flow. Each stage uses a stack of mesoporous mixed-cellulose-ester (MCE) membranes (Millipore, MA, USA) as the medium for thermal transpiration^33^. Each membrane has an average pore diameter of 25nm, a porosity of 70%, and a thickness of 100 μm. The stack has a total thickness of 500 μm and a footprint of 16×16 mm^2^. The membrane stack is sandwiched by three glass dies on each side. The two inner glass dies contain flow channels, and the two middle glass dies contain embedded thin-film metal heaters and thermistors, whereas the two outer glass dies provide common anchor planes (Figure 1). Commercially available fin-shaped heat sinks are attached to the external surfaces of the outer glass dies. Because the flow is directed from the unheated side to the heated side, having the heaters embedded on both sides of the membrane stack allows the flow direction to be reversed when switching from sampling to separation.

In the present manifestation of the overall system, all the fluidic components are arranged in a planar manner in both the chromatography module and the Knudsen pump module. Whereas the fluidic connection between the two modules uses conventional tubing to facilitate modular reconfigurability, within each module, the components are connected by co-fabricated gas flow connectors. The short length of these connectors (7 mm) permits compact arrangement of the components, minimizing the overall footprint.

### Fabrication

We previously reported a three-mask process for fabricating various μGC components^16^. In that process, a glass wafer was first metallized and subsequently micromachined. In the current study, metallization and micromachining were performed on different glass substrates that were later bonded together. Consequently, the micromachining and metallization steps were not performed sequentially. Although the new approach cannot be used for stacked architectures^16,17^ that require through holes to provide vertical gas flow paths, it is feasible for the planar arrangement of components described in this study. The basic elements of each of the system components are fabricated from glass wafers by a common three-mask process. One mask is used to pattern a thin film of Ti/Pt (25/100 nm) for the heaters, thermistors and capacitor electrodes. The other two masks are used for sandblasting (available as a service from Bullen Ultrasonics, Inc., OH, USA). One mask defines gas flow channels that are sandblasted to a depth of 250 μm on one side of a glass wafer; the other mask defines through perforations that are sandblasted from the other side of the wafer to make chip-to-chip fluidic connections. The sandblasting process typically creates a 22° sidewall taper angle and a 125 μm corner radius^16^.

The Knudsen pump is assembled by sandwiching the MCE membrane stack within the microfabricated glass dies and sealed with an epoxy (Stycast2850FT, Henkel, Germany). The MCE membrane and the epoxy can potentially introduce mild outgassing issues that interfere with the chromatographic separation; these issues can be addressed by proper system operation, as discussed in the experimental section. The preconcentrator is assembled by bonding the glass dies with another epoxy (Epotek-377, Epoxy Technology Inc., MA, USA), followed by baking at 300°C for >1 h to minimize outgassing during operation. (Additional purges can be performed at lower temperatures even after the assembly of the complete system.) Two types of sorbent particles, that is, Carbopack-X and Carbopack-B, as well as glass beads, are sequentially packed by a gentle vacuum into the 'U' channel from a loading port that is subsequently sealed with Stycast2850FT epoxy. The separation column is assembled by bonding the glass dies with a spin-coated layer of adhesion material (SU-8, MicroChem, MA, USA), followed by the application of an OV-1 stationary phase coating to the interior of the column using the established static coating method^35^.

For the capacitive detectors, the metallized substrate is coated with a 10-nm-thick Al _2_O_3_layer using atomic layer deposition. This is an optional, precautionary step to prevent potential shorting caused by dust; that is, the step can be excluded if the assembly is performed in a clean environment. Next, an OV-1 solution is spin-coated onto the metallized substrate. The OV-1 solution is prepared by dissolving OV-1 in nonane. A small quantity of dicumyl peroxide (<10% of OV-1 in weight) is also added to the solution to induce cross-linking^35^. Other common non-polar solvents such as pentane and hexane have also been evaluated for preparing the solution. However, these solvents result in randomly located cavities in the OV-1 layer, possibly because these solvents are highly volatile and form bubbles during spin-coating. In contrast, the less volatile nonane provides a smooth and uniform OV-1 film after spin coating. Subsequently, the OV-1-coated substrate is bonded to the sandblasted substrate with epoxy.

**Figure 2:**
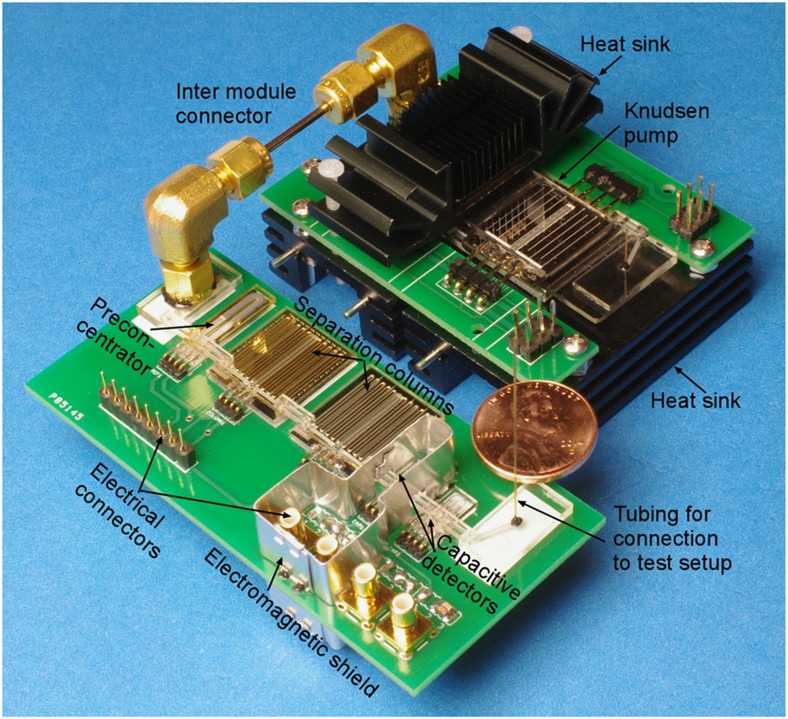
Photograph of the *iGC3.c2* system. One of the Knudsen pump heat sinks has been removed to reveal the inner structure.

The fluidic components of the chromatography and pump modules are mounted on separate printed circuit boards (PCBs). The microfabricated gas flow connectors are connected between the fluidic components. In the current manifestation of the system, commercial pins and headers are directly soldered to the components to provide electrical lead transfer between the components. To prevent on-board electromagnetic crosstalk that can affect the detector readout, CapDet1 is fenced in by an aluminum foil perimeter that is grounded. A photograph of the system is shown in Figure 2. The overall footprint of the system, including both modules, is 8×10 cm^2^.

### Test vapor preparation

To evaluate its performance, the *iGC3.c2* system was used to sample, separate and detect mixtures of 19 chemicals. The chemical species were labeled in groups based on their molecular structures or properties. The *a*-group contained six non-polar *n*-alkanes ranging from pentane (C_5_) to decane (C_10_); the *b*-group contained five non-polar aromatic hydrocarbons: benzene, toluene, *m*-xylene, *o*-xylene and mesitylene; the*c*-group contained five mildly polar chemicals (aldehyde and halogenated hydrocarbons): hexanal, chlorobenzene, chlorohexane, 4-chlorotoluene and 1,3-dichlorobenzene; and the *d*-group contained three other non-polar chemicals (cycloalkane and terpenes): cycloheptane, α-pinene, 3-carene. Most of these chemicals are common indoor air pollutants^2^.

To prepare the test vapor, the 19 chemicals in liquid form were first formulated into a mixture. The volume of each chemical was adjusted (based on density and molar mass) such that the concentrations of the non-polar chemicals were equal in the resulting vapor and the concentration of each mildly polar chemical was 1/5 of that concentration. For mixtures that presented relatively high levels of concentration (in which each non-polar chemical had a concentration of 0.5-1 p.p.m.), liquid mixtures with volumes of 85-170 nl were injected into a 2-l dilution bottle prefilled with room air. For mixtures that presented lower concentrations, the injected liquid volume became difficult to control accurately. Therefore, these liquid mixtures were diluted with pentane before being injected into the 2-l bottle. In each case, the mixture injected into the bottle evaporated completely and formed the test vapor. Table 1 summarizes the vapor concentrations that were tested. In this paper, the units for vapor concentration, i.e., 'p.p.m.' and 'p.p.b.', are defined as the number of molecules of each chemical divided by the number of molecules of air in the prepared vapor, multiplied by 10^6^ and 10^9^, respectively.

### Test method

**Figure 3:**
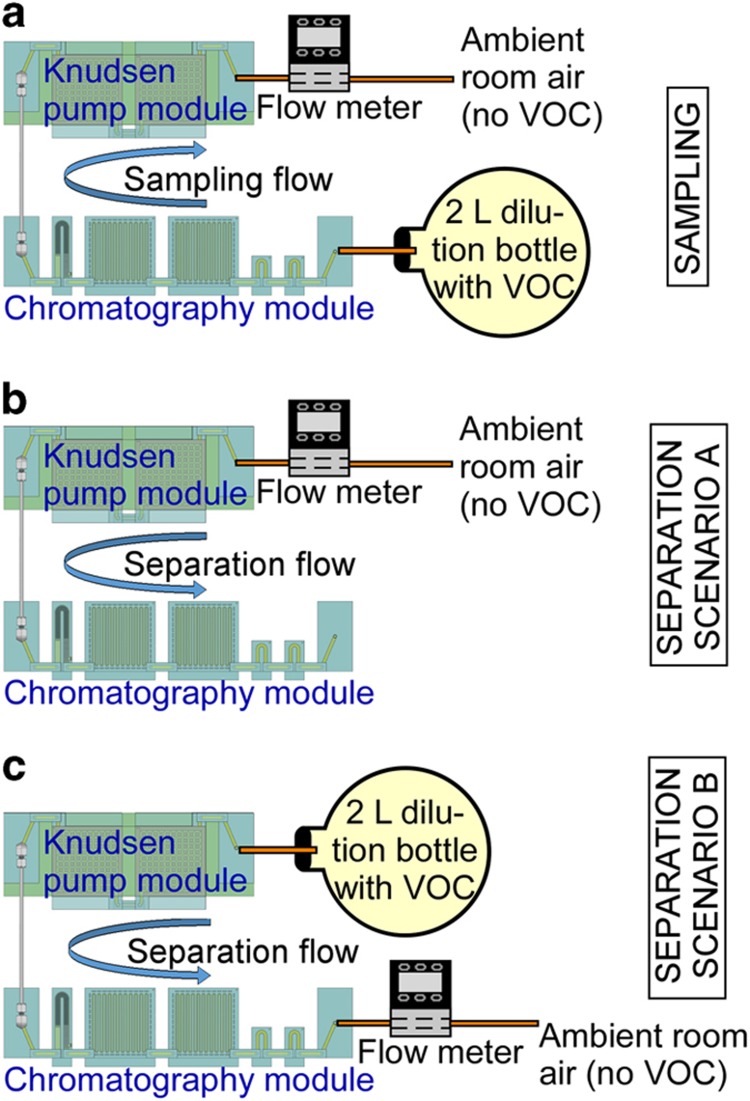
Test setup for emulating two scenarios of system operation. Scenario A: a conventional scenario in which vapor samples are manually collected and fed to the system; the carrier gas is room air without the analytes. Scenario B: an emerging scenario in which the system automatically monitors the ambient pollutants; the carrier gas contains the analytes. (**a**) Test setup for vapor sampling. (**b**) Test setup for emulating analytical separation in Scenario A. (**c**) Test setup for emulating analytical separation in Scenario B. VOC, Volatile organic compound.

During the vapor sampling stage of each experiment, when the sample VOCs were accumulated in the preconcentrator, the bottle was connected to the port upstream of the capacitive detectors (Figure 3a). The vapor was drawn from the bottle through the detectors and the separation columns into the preconcentrator by the Knudsen pump module, which was operated at 0.2 sccm for 10-120 min. The temperature in the pump was controlled by the on-chip heaters and temperature sensors operated by a customized closed-loop control algorithm in LabVIEW, which used pulse-width modulation (PWM) to control the heater power. As previously reported^16^, the output flow rate of the Knudsen pump is roughly proportional to the temperature difference across the MCE membranes (Δ*T*) and it is also dependent upon the average temperature of the MCE membranes (*T*_avg_). For the experiments reported in this study, the flow rate could be determined by setting either Δ*T* or the ratio Δ*T*/*T*_avg_; both provided stable control of the flow rate. If*T*_avg_ varied widely, then a more complex function consisting of Δ*T* and *T*_avg_ could be used in the PWM algorithm. The pump consumed 3.2 W during vapor sampling. The power estimate accounts for the experimentally measured average voltage and current generated by the PWM controller during this period. A commercial flow meter (Model # MW-20SCCM-D, Alicat Scientific, Inc., Tuscon, AZ, USA) was optionally connected downstream of the pump to provide readings of the flow rate. The other end of the flow meter was open to room air. The control of the system did not rely on the flow meter. The chromatography module was maintained at room temperature. After vapor sampling, the Knudsen pump was turned off and allowed to cool down for 15-25 min before initiating analytical separation.

**Figure 4:**
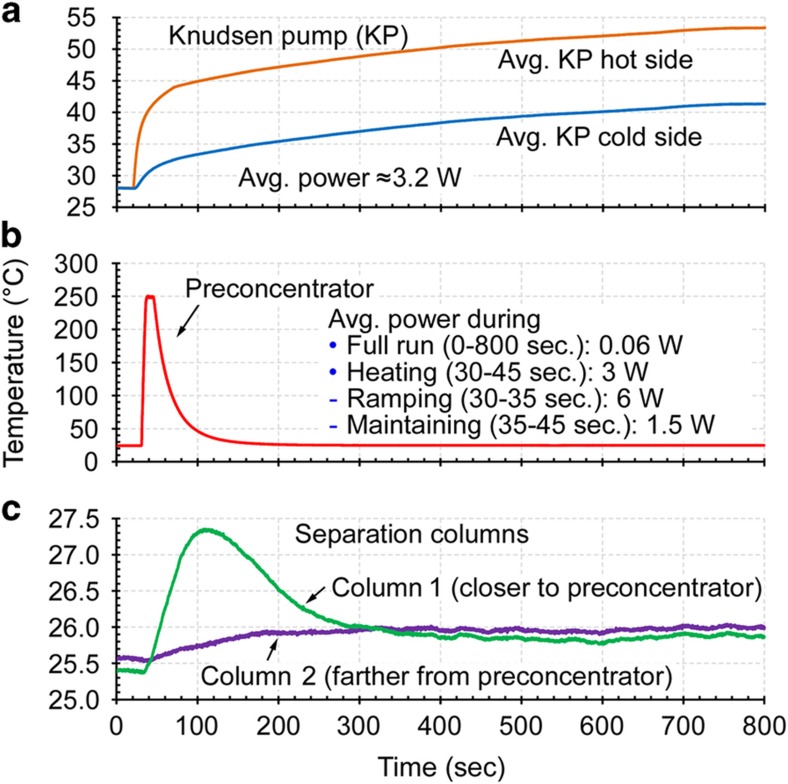
Typical temperature control of the system during analytical separations without active temperature programming of the separation columns. (**a**) The temperatures at the Knudsen pump. Continuous temperature difference between the hot side and cold side provides continuous flow. (**b**) The preconcentrator temperature during a typical thermal pulse to perform vapor injection. (**c**) The temperature rise of the separation columns resulting from thermal crosstalk with the preconcentrator.

During the analytical separation stage of each experiment, the dilution bottle was removed. As a result, room air served as the carrier gas in the separation stage (Figure 3b).This arrangement emulated the conventional application scenario in which vapor samples are manually collected and injected to the system. An alternative scenario was also emulated: autonomous operation in which polluted air containing the analytes serves as the carrier gas during separation (Figure 3c); this scenario is described in a separate subsection. The moment at which the software sequence for control and readout was initiated was defined as time *t*=0. Starting at time *t*≈20 s, the pump provided a reversed 0.2-sccm flow (using 3.2 W), pushing room air without analytes into the chromatography module. After the flow rate reached the appropriate level, starting at time *t* ≈30 s, the preconcentrator was heated to 250 °C in 5 s and maintained at this temperature for 10 s (Figure 4) to thermally inject the sampled vapors into the closest separation column (Column 1). Like the Knudsen pumps, the preconcentrator was heated using the embedded thin-film metal heaters, and its temperature was monitored using embedded thermistors. Even though temperature programming of the separation columns was evaluated as described in the next section, for most experiments the columns were not actively heated. For separation columns of relatively short length, the selected set of VOC indoor pollutants provided modest elution times, reducing the need for temperature programming. In experiments that were performed without deliberately heating the separation columns, Column 1 experienced a temporary temperature increase of 2 °C due to crosstalk from the thermal pulse delivered to the preconcentrator, whereas Column 2 experienced an increase of about 0.5 °C (Figure 4).

**Figure 5:**
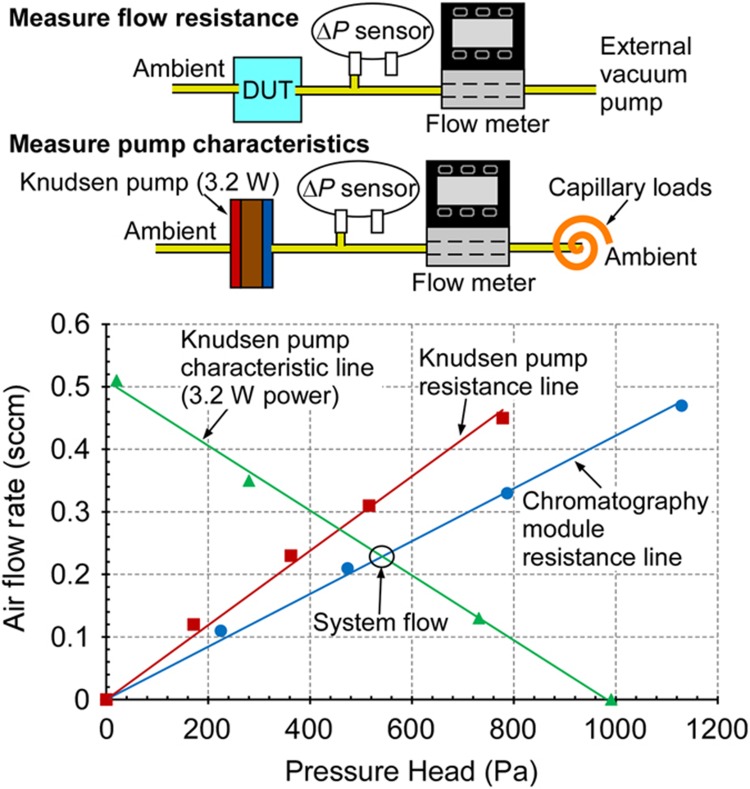
Pump characteristics and flow resistance of the Knudsen pump module and chromatography module.

During either sampling or separation, the 0.2-sccm air flow through the system resulted from the overall pressure load of the fluidic path presented to the Knudsen pump module (Figure 5). The flow properties of the two modules were measured in separate experiments. The flow resistance of the chromatography module was measured by using an external vacuum pump to create an air flow through the module, whereas the flow rate and pressure difference along the module were measured by a flow meter (MW-20SCCM-D, Alicat Scientific, Inc.,) and pressure sensor (MPX5010DP, Freescale Semiconductor Inc., Austin, TX, USA), respectively. The flow resistance of the Knudsen pump module was measured in the same manner. The output pressure head and flow rate characteristics of the Knudsen pump module were measured at 3.2 W. The pump provided a maximum flow rate of 0.5 sccm and a blocking pressure of 1 kPa. The intercept of the pump characteristic line and the chromatography module resistance line appeared at 0.2 sccm, as was experimentally confirmed by the flow meter.

The two capacitive detectors were simultaneously measured by two commercial 24-bit capacitive-to-digital converter (CDC) chips (AD7746, Analog Devices, Norwood, MA, USA). Available CDC options offering lower resolution (for example, 16 bit) were not in order to prevent electronic noise from contributing to the observed VOC detection limits. Each CDC provided a square-wave excitation signal to one set of electrodes in the capacitive detector and measured the voltage from the opposite set of electrodes. Because the excitation signal for one detector could affect the voltage readout from the other detector through electromagnetic coupling, an electromagnetic shield, made of custom-cut aluminum sheets and connected to the CDC ground, was placed on the PCB, as noted in 'Materials and Methods'. The CDCs used a detection rate of 6-7 Hz. When the CDC was not connected to any capacitor, its rated value and measured value of root-mean-square (RMS) noise were both 4 aF. When the CDC was connected to the capacitive detector in the current setup, the measured RMS noise was 40 aF. The noise increase could potentially be reduced with improved PCB design. For all the experiments performed in this work, the capacitive detectors were operated at an ambient temperature of 22 °C.

A number of experiments were performed on test samples, as explained in the next section. Before each analytical cycle, the preconcentrator was flushed for 35 s by providing the type of thermal pulse used for the vapor injection, while the pump provided 0.2-sccm flow in the direction used for vapor sampling. This procedure prevents the accumulation of contaminants in the preconcentrator from run to run, essentially regenerating the system; it could be particularly helpful when using polluted air as the carrier gas.

## Results

### Quantitative analysis

**Figure 6:**
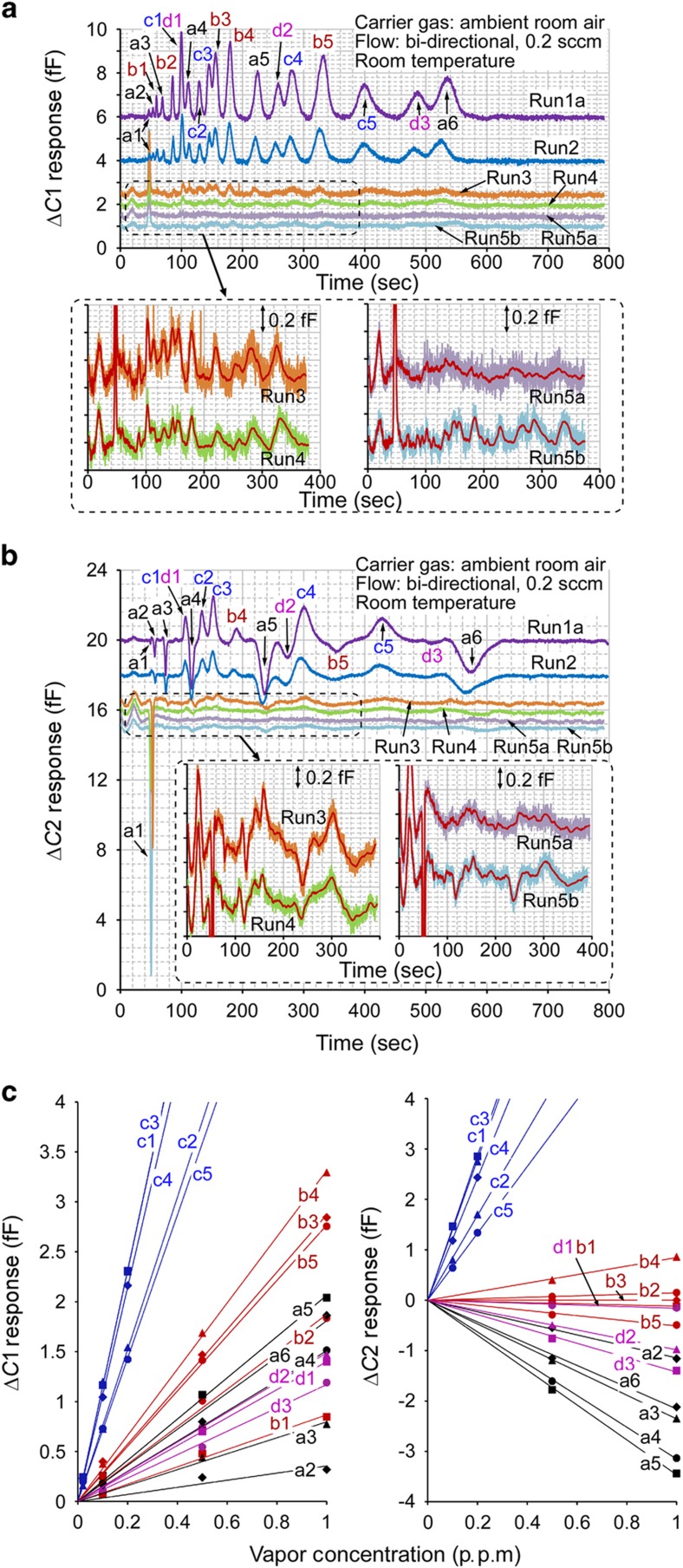
*iGC3.c2* system results for 19 chemicals with varying concentrations. Separations were performed at room temperature. (The chromatograms are shifted vertically for visual clarity). (**a**) Chromatograms provided by CapDet1. (**b**) Chromatograms provided by CapDet2. (**c**) Measured peak heights (Δ *C*1 and Δ*C* 2) showing proportionality to prepared vapor concentrations. p.p.m., parts per million.

To demonstrate quantitative analysis, the system was used to evaluate the 19-chemical mixture (Table 1). The chromatograms obtained from CapDet1 and CapDet2 are shown in Figures 6a and b, respectively. A sampling time of 30 min was sufficient to demonstrate the effective separation and detection of chemicals with concentrations ranging from 0.05 to 1 p.p.m. for non-polar species and from 0.01 to 0.2 p.p.m. for mildly polar species (for example, in Runs 1-4, Figures 6a and b). For lower concentrations (that is, 10 p.p.b. for non-polar species and 2 p.p.b. for mildly polar species), the 30-min sample did not provide distinguishable peaks (for example, in Run 5a). However, a sampling time of 2 h did provide distinguishable peaks (for example, in Run 5b, insets of Figures 6a and b).

### Data processing

Baseline variations in raw chromatograms were digitally compensated by a customized MATLAB program. First, baseline segments were identified in the chromatogram; this was performed manually in the present work, but in principle can be performed automatically by software^36^. Next, a polynomial was used to fit the baseline segments and was then subtracted from the raw chromatogram.

The chromatograms were de-noised by a progressive moving average method. Moving averages are typically used to process transient data streams: a fixed-length window moves along the stream, and raw data within the window are averaged to generate a new data point. The window must be long enough to minimize the noise in the data yet small enough to preserve the signal intensity. In the case of GC chromatograms, later retention peaks are typically wider than earlier ones because of diffusion in the separation columns^8^. Therefore, the GC chromatograms are amenable to the calculation of a moving average with an increasing window length. For the chromatograms of Runs 3-5 (in the insets of Figures 6a and b), the window length for the chromatograms was linearly ramped from 5 points (before *t*≈30 s) to 101 points (at the end of the chromatograms). This method clearly reduces the noise without significantly distorting the retention peaks.

### Linearity and sensitivity trends

The peak heights extracted from the chromatograms varied proportionally with the corresponding vapor concentrations being sampled (Figure 6c). This proportionality is expected for capacitive detectors. Compared with detectors that provide linear response in log or log-log scales^12,24^, the directly proportional response of the capacitive detectors can be more readily interpreted. Furthermore, as indicated by the slopes in Figure 6c, both CapDet1 and CapDet2 showed higher sensitivity to the *c*-group (mildly polar) chemicals than to the *a*-, *b *- and *d*-group (non-polar) chemicals. Compared with CapDet1, which showed positive peaks for all the tested chemicals, CapDet2 showed positive peaks for the *c*-group, negative peaks for the *a*- and *d *-group, and nearly zero response to the *b* -group. In addition, both detectors showed a common trend for the sensitivity to alkanes: the absolute value of sensitivity increased from pentane (*a1*) to nonane (*a5*) but decreased from nonane to decane (*a6*). These observations are discussed in the next section.

### Resolving co-eluting peaks

**Figure 7:**
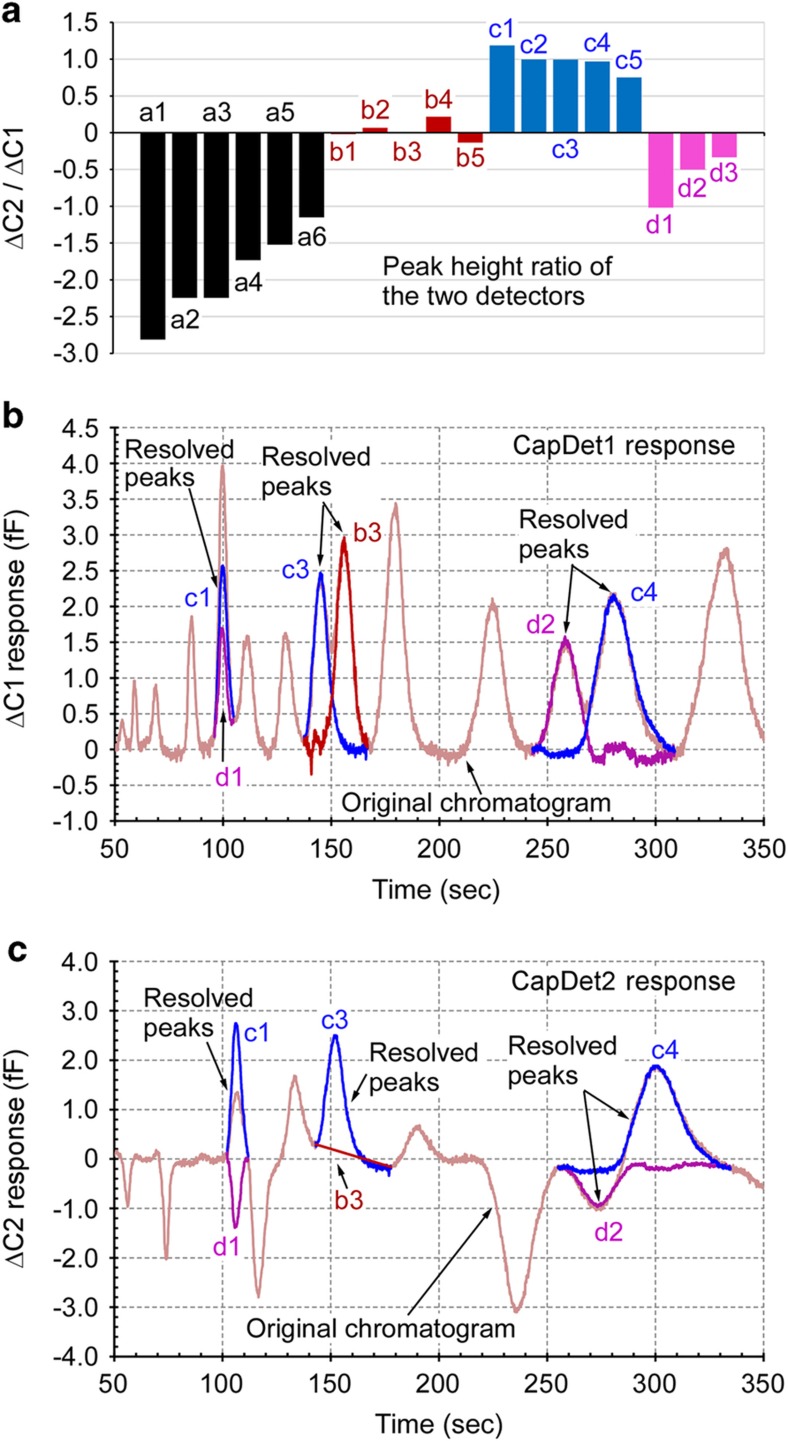
Co-eluting peaks resolved by the two capacitive detector responses. (**a**) Capacitive detector response ratios (Δ*C*2/Δ*C* 1) for various chemicals. (**b**) Co-eluting peaks resolved in CapDet1 chromatogram. (**c**) Co-eluting peaks resolved in CapDet2 chromatogram.

In some examples of μGC systems, arrays of detectors, each having a unique response pattern to various chemicals, have been used to enhance the discrimination of eluted peaks^13,26,37^. In contrast to the reported chemiresistor arrays^37^, which provide only positive peaks to almost all chemicals, capacitive detectors can provide both positive and negative peaks ^27,30^, thereby providing higher discrimination capabilities for certain chemical species. In this study, the *a*- and *d*-group chemicals in particular could be easily differentiated from the *c*-group chemicals using CapDet1 and CapDet2. In addition, these two detectors could be used to resolve co-eluting peaks. To resolve these peaks the detector response ratio, i.e., *r*=Δ*C*2/Δ*C*1, was first measured for each chemical (Figure 7a). These measurements were performed in two sets, with each set including chemicals that did not produce co-eluting peaks. (Additional sets may be necessary in other cases.) In a chemical mixture that contains two co-eluting species, *A* and *B*, the following equation matrix can be formulated:

(1){ΔC1A+ΔC1B=ΔC1rA . ΔC1
A+rB . ΔC1B=ΔC2

where Δ*C*1_*A*_ and Δ*C*1_*B*_ are the contributions of chemicals *A* and *B* to the peak obtained from CapDet1, and *r*_*A*_ and *r*_*B*_ are the respective response ratios. By solving this equation matrix for every data point in the chromatogram segments containing the two co-eluting peaks, each co-eluting peak can be separately reconstructed. As shown in Figures 7b and c, this method successfully resolves partially co-eluting peaks *c3*-*b3* and *d2*-*c4*, as well as fully co-eluting peaks *c1*-*d1*.

### Temperature programming

**Figure 8:**
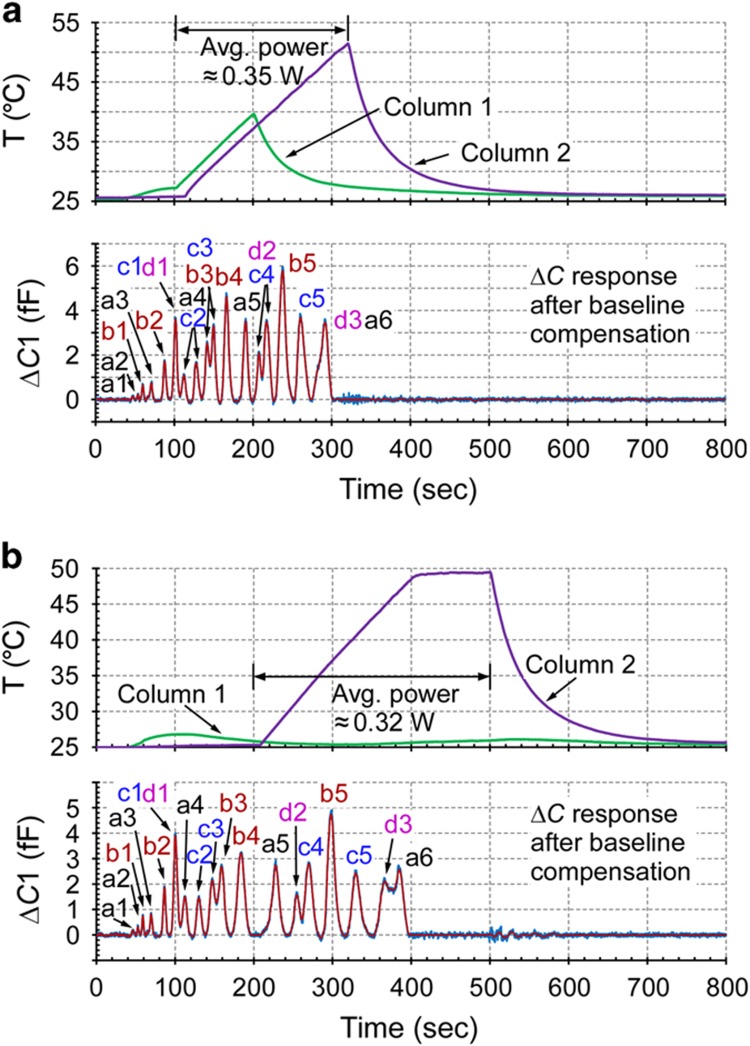
Accelerated separation provided by temperature programming of the separation columns. (**a**) Run 1b: similar to Run 1a in Figure 6 but with both columns heated for fast separation. (**b**) Run 1c: heating only Column2 to accelerate separation with lower power consumption.

For microfabricated separation columns, each column chip can often be individually programmed^13^. Two options were evaluated: with both separation columns actively heated independently (Figure 8a) and with only the downstream column heated (Figure 8b). In the former case, in Run 1c (Figure 8a), the temperature of Column 1, which was upstream of Column 2 during separation, was ramped linearly from room temperature to 40 °C from *t*=100 s to *t*=200 s and then allowed to cool naturally. Further heating of Column 1 was unnecessary because all the tested chemicals had eluted Column 1. The temperature of Column 2 was linearly ramped to 52 °C from *t*=100 s to *t*=320 s and then allowed to cool naturally. Compared with room-temperature separation, this temperature programming run accelerated the separation by 50% and increased the peak heights of certain chemicals by 50-100%. The columns consumed a total average power of 0.35 W from *t*=100 s to *t*=320 s. The power consumption can be slightly reduced by heating only the downstream column. In the latter case, in which only the downstream column was heated, in Run 1d (Figure 8b), the temperature of Column 2 was linearly ramped to 50 °C between 200 and 400 s. Column 1 was not actively heated but may have been passively heated by a few degrees because of thermal crosstalk. Compared with room-temperature separation, this temperature programming run accelerated the separation by 30%, while the columns consumed only 0.32 W. The energy consumption could be further reduced by stopping the heating of Column 2 at the end of the ramp (Figure 8b).

### Ambient VOC in carrier gas

The aforementioned experiments used the ambient room air as the carrier gas, which was relatively clean and not contaminated by the analyte samples. These results demonstrated the operation of the system in a conventional test scenario, in which vapor samples are manually collected and fed to the system for analyses. However, if the system is operated in a different scenario in which the ambient pollutants are being monitored automatically, then the carrier gas used for analytical separation would be polluted by the same vapor being sampled.

**Figure 9:**
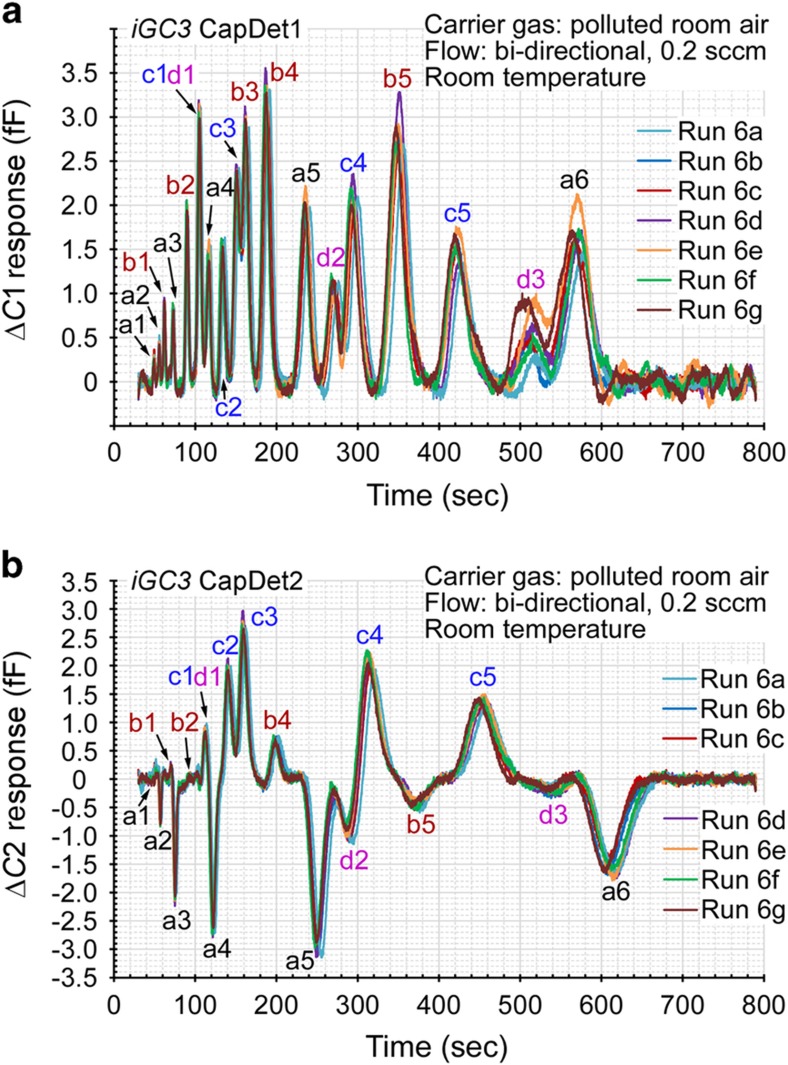
Chromatograms of repeated runs using both ambient and polluted air as the carrier gas. Run 6a used the (relatively clean) ambient air as the carrier gas. Run 6b-c used polluted air with 10-25% ambient relative humidity as the carrier gas for benchmarking. Run 6d-g used polluted air with 50% relative humidity as the carrier gas. (**a**) CapDet1 response. (**b**) CapDet2 response.

To mimic this latter scenario, another set of experiments was performed, in which the dilution bottle filled with the tested vapor mixture was connected upstream of the Knudsen pump during analytical separation to provide the carrier gas (Figure 3c). In principle, the preconcentrator, after thermal desorption, traps the VOCs in the carrier gas, preventing the VOCs from entering the separation columns. Therefore, in a single run, the results obtained using polluted air as the carrier gas should be similar to those obtained using ambient air. In consecutive runs, however, the VOCs trapped by the preconcentrator during previous analytical separation could be injected, together with the new sample, into the separation column in the following run, causing hysteresis and overestimation of the VOC concentrations. Therefore, before each vapor sampling cycle, the preconcentrator was flushed by flow in the direction of sampling for a short duration. In experimental evaluation, the system was tested by performing consecutive runs. As demonstrated by the experimental results in Figure 9, the chromatograms for the 19 chemicals (1 p.p.m. in the *a*-, *b*- and *d*- groups, 0.2 p.p.m. in the *c*- group) in the carrier gas (Run 6b-g) overlap with the chromatogram obtained using the relatively clean ambient air as the carrier gas (Run 6a). These results indicate that the system can perform repeatable analyses in a polluted environment. 

### Effect of humidity

The relative humidity in the laboratory where the tests were conducted varied between 10 and 20%, as measured by a commercial humidity sensor (#42280, Extech Instruments Corporation, Nashua, NH, USA). To test the system response to moisture, a volume of water was injected into the 2-l dilution bottle together with the 19 VOCs such that the relative humidity in the sampled vapor was 50%, which is equivalent to a concentration of 12 000 p.p.m.. The increased moisture caused baseline variations. However, after baseline compensation, the system response to the moisturized vapor (Run 6d-g) overlapped with that for the non-moisturized vapor (Run 6a-c, Figure 9).

## Discussion

The results of the two capacitive detectors warrant discussion. As noted previously, the Δ*C* depends on changes in both thickness and permittivity that are caused by the eluted peaks of the VOCs in the OV-1 polymer film covering the electrodes. The permittivity response can be expressed as the difference in the dielectric constants of the polymer and chemical, i.e., *ε*_ch _-*ε*_p_, in the affected region of OV-1. If this term is positive, then the average dielectric constant of the polymer-chemical matrix is higher than that of the polymer, and the resulting Δ*C* will also be positive. Conversely, if this term is negative, then its contribution to the Δ*C* will be negative.

In CapDet2, where the polymer thickness of 1.6 μm is greater than the electrode width and gap of 1 μm, polymer swelling does not affect Δ*C* because most electric field lines for sensing are distributed only in the lower portion of the polymer. Hence, Δ*C*2 depends almost entirely on *ε*_ch_-*ε*_p_ within the fixed volume traversed by the field lines. The dielectric constant of OV-1 (*ε*_p_), which is composed of 100% polydimethylsiloxane or PDMS, at room temperature is 2.3-2.8 (ref. 38), whereas the dielectric constants of the *a*-, *b*-, *c*- and *d*- group chemicals (*ε*_ch _) are 1.8-2.0, 2.2-2.6, 5-10 and 2.0-2.2, respectively^39^. It should be noted that the dielectric constants of chemicals under consideration are the values for the liquid phase rather than for the gas form because the vapor molecules adsorbed in the polymer are condensed. Therefore, the *c*- group contributes to a positive Δ *C*2 value, the *b* group contributes to a nearly zero Δ*C*2 value and the *a* and *d* groups contribute to a negative Δ*C*2 value (Figure 6b).

In CapDet1, the polymer thickness of 0.25 μm is substantially smaller than the electrode width and gap of 1 μm. Therefore, polymer swelling does affect Δ*C*1 because the entire polymer thickness is within the distribution of electric field lines for sensing. Polymer swelling always produces a positive Δ*C*1 because an increasing number of electric field lines traverse the polymer. The VOC molecules that cause the swelling of the polymer layer also contribute dipole moments that add to the capacitive response. As demonstrated by the results (Figure 6a), Δ*C*1 is positive for all the tested chemicals, even for the *a*-group chemicals that provide negative *ε*_ch_-*ε*_p_. In addition, Δ*C*1 is strongly dependent upon the value of *ε*_ch _-*ε*_p_. The *c*-group chemicals provide values of *ε*_ch_-*ε*_p_, that are several times higher than those provided by non-polar chemicals, and hence the values of Δ*C*1 are also several times higher.

The sensitivity of the capacitive detectors also depends on the partition coefficients (*K*_D_) of chemicals between the polymer and air. The partition coefficient is defined as the ratio—at equilibrium—of the chemical concentration in the polymer to that in air. This measure is used to describe the extent of vapor adsorption in the polymer. The partition coefficient multiplied by the phase ratio (*β*), that is, volume ratio of the air to the polymer in the detector, can be used to determine the fraction of chemicals (*η*) adsorbed by the polymer and useful for sensing^8^:

(2)η=KD/βKD/β+1

On one hand, large *K*_D_ values produce large *η* values and hence increase the magnitude of Δ*C*. On the other hand, because the separation columns use the same polymer as the detectors in the *iGC3.c2* system, larger *K*_D_ values cause longer retention and broader peaks, thereby reducing the magnitude of Δ*C *. The *β* values for CapDet1 and CapDet2 are 1000 and 160, respectively. The partition coefficients of the *a*-group chemicals between PDMS and air at room temperature^40^, as well as the calculated values of *η*, are listed in Table 2. From pentane to nonane, the *η* value substantially increases (Table 2), causing the magnitude of Δ*C* to increase in both detectors (Figure 6). Compared with nonane, decane yields only marginally larger *η* values but much broader peaks, hence providing slightly smaller Δ*C* magnitudes in both detectors (Figure 6). Similar trends are also evident for the *b*-group chemicals in the CapDet1 responses (Figure 6 ). It is worth noting that the partition coefficient describes the chemical distribution only when equilibrium is reached. Because of the continuous flow that exists in a μGC detector, the actual fractions of chemicals adsorbed by the polymer are possibly smaller than the calculated values shown in Table 2. A more detailed understanding of the dynamic distribution of the chemical molecules can be obtained with computational modeling^27^.

Further calculations can be performed to elucidate the chemical quantities being analyzed. For example, in Run 5b, the 120-min sample taken at a flow rate of 0.2 sccm provided a sampling volume of 24 ml. Hence, the mass of chemicals being sampled and subsequently injected by the preconcentrator was 1 ng for the non-polar chemicals and 0.2 ng for the mildly polar chemicals. (The effective mass actually sampled into the preconcentrator was slightly smaller because of the threshold volume, as discussed in the following text; nevertheless, this difference was negligible for the 120-min sample.) These values represent the lowest mass levels that were experimentally detected by the capacitive detectors.

In addition, in this study, considering 4-chlorotoluene as an example, the peak width (measured at half peak height) was 17 s. The gas flow rate was 0.2 sccm during separation and hence this species occupied a volume of 0.06 ml. Therefore, the maximum concentration of the 4-chlorotoluene peak was 0.8 p.p.m. because 24 ml of the original 2 p.p.b. vapor was concentrated in the 0.06 ml peak. The *K*_D_ of 4-chlorotoluene, as calculated based on a previously reported formula^40^, is 5590. Thus, only 15% of the 4-chlorotoluene molecules remained in the gas phase during the time that the peak passed through CapDet1. If the detector had been tested in a non-transient steady-state gas environment with a large head space, a concentration of 120 p.p.b. of 4-chlorotoluene would be necessary to produce the same reading. This type of correction is necessary when comparing the *iGC3.c2* results with those in which chemical sensors are characterized at steady state.

In this study, the capacitive detectors used OV-1 as the polymer film. Being non-polar, this material is most responsive to non-polar and mildly polar chemicals. For highly polar chemicals, a polar polymer film is recommended. Polar chemicals inherently provide higher values of *ε*_ch_-*ε*_p_ than chemicals with lower polarities and are likely to provide even lower detection limits ^25,26^.

It is notable that the detection principle of the interdigitated capacitive detectors can be modeled^27,30^, and hence a library of predictable detector responses can be constructed using computational methods. Such a library can reduce the burden of evaluating the technology for a previously untested application.

In the *iGC3.c2* system, the sampled vapor must pass through the detectors and columns before reaching the preconcentrator, requiring a threshold sampling duration to be exceeded for the preconcentrator to receive sampled vapor. This threshold sampling duration can be measured at any vapor concentration by plotting the variation in the output with sampling duration. For the *iGC3.c2* system with a flow rate of 0.2 scm during sampling, the threshold sampling durations for *m*-xylene, 4-chlorotoluene and decane are 4, 7 and 10 min, respectively.

The use of ambient air as the carrier gas eliminates the need for a bottled carrier gas, which is a consumable resource and an additional hardware item. The use of air is attractive for applications that require extended autonomous operation. For a given separation column, the highest achievable separation efficiency provided by air as a carrier gas is similar to that provided by the more commonly used nitrogen or helium^41^. However, to achieve the highest efficiency, the required flow rate of air is less than that of helium^41^. For high-speed separations in which the flow rate often exceeds the value required to achieve the highest efficiency, helium provides higher efficiency than air^41^ because helium enables faster mass transfer of VOCs between the carrier gas and the stationary phase. In contrast, for analyses in which speed is not a primary concern, the flow rate can be set to the value required to achieve the highest efficiency.

The use of air as the carrier gas can potentially cause PDMS degradation (manifested as weight loss) at elevated temperatures. Because this polymer is used both as the stationary phase in the separation columns and the sensing layer in the capacitive detectors, this degradation can be a significant concern. However, the literature shows that thermal oxidative degradation starts to become significant only above 290 °C^42^. In experiments, a typical *iGC3.c2* system was operated at room temperature (22 °C) intermittently for <50 runs over a period of 4 months without showing a decrease in the VOC retention times or the capacitances of the detectors, both indicating no PDMS weight loss. In temperature programming experiments that were performed in this study, the maximum temperature in the separation columns was only approximately 50 °C.

In conclusion, the results show the viability of a full μGC system in which all the components are microfabricated and electronically interfaced. The system has no moving parts and uses room air as the carrier gas, and all the fluidic components can be co-fabricated by a common low-cost process. These features can potentially increase the overall applicability of μGC technology. The current manifestation of the system provided a detection limit of 2 ppb for certain chemicals, which is low enough for use in applications such as indoor pollutant monitoring. Compared with other reported μGCs that provide comparable performance^12,13^, the results of this study are significant because they are provided by a fully microfabricated and integrated system. With further refinement of the electronic hardware and software, the system is expected to be suitable for continuous, unmanned monitoring.

In the future, the system platform can be reconfigured for other applications. For example, detectors with different coatings of polymer species can be easily integrated to enhance vapor recognition. More preconcentrators or separation columns can be added to enhance the chromatography performance. Whereas in the current system a two-stage Knudsen pump is used to demonstrate a scalable architecture, a single-stage Knudsen pump with improved design and implementation is also expected to be capable of driving the system, thereby reducing the system complexity and footprint. Other micro gas pumps can also be integrated in place of the Knudsen pump module. Planned future work also includes the comprehensive characterization of all components—particularly the Knudsen pump, separation column, and capacitive detectors—operating at various ambient temperatures, which will establish a database for field use.

## Figures and Tables

**Table 1:** List of chemicals and test conditions

Run ID:	1		2	3	4	5	
	1a	1b*, 1c*				5a	5b
Sampling time (min)	30						120
Chemicals	Concentration (p.p.m.)						
*a *1	1		0.5	20	10	10	
*a*2-*a*6	1		0.5	0.1	0.05	0.01	
*b*1-*b*5	1		0.5	0.1	0.05	0.01	
*c*1-*c*5	0.2		0.1	0.02	0.01	0.002	
*d*1-*d*3	1		0.5	0.1	0.05	0.01	

*a*1-*a*6: *n*-alkanes C_5_-C_10_ (nonpolar).

*b*1-*b*5: benzene, toluene, *m*- and *o*-xylene, mesitylene (nonpolar).

*c* 1- *c* 5: hexanal, chlorobenzene, chlorohexane, 4-chlorotoluene, 1,3-dichlorobenzene (mildly polar).

*d* 1- *d *3: cycloheptane, Î±-pinene, 3-carene (nonpolar).

Relative humidity: 10-20%.

* Column temperature ramped during separation for Runs 1 *b *, 1*c *.

**Table 2:** Calculation of chemicals partitioned between polymer and air in the detectors

VOC chemicals	Partition coefficient (*K*_*D*_)^40^	Fraction of chemical in polymer in CapDet1 (*η*1) (%)	Fraction of chemical in polymer in CapDet2 (*η*2) (%)
Pentane	82	8	34
Hexane	215	18	58
Heptane	565	36	78
Octane	1486	60	90
Nonane	3908	80	96
Decane	10 280	91	99
Benzene	296	23	65
Toluene	815	45	84
*m * -Xylene	2190	69	93
*o * -Xylene	2710	73	95
Mesitylene	6150	86	98
